# Borderline Personality Features in Patients With Persistent Depressive Disorder and Their Effect on CBASP Outcome

**DOI:** 10.3389/fpsyt.2021.608271

**Published:** 2021-03-12

**Authors:** Franziska Konvalin, Fabienne Grosse-Wentrup, Tabea Nenov-Matt, Kai Fischer, Barbara B. Barton, Stephan Goerigk, Eva-Lotta Brakemeier, Richard Musil, Andrea Jobst, Frank Padberg, Matthias A. Reinhard

**Affiliations:** ^1^Department of Psychiatry and Psychotherapy, LMU University Hospital Munich, Munich, Germany; ^2^Department of Clinical Psychology and Psychotherapy, University of Greifswald, Greifswald, Germany

**Keywords:** persistent depressive disorder, borderline personality disorder, comorbidity, CBASP, childhood maltreatment, rejection sensitivity

## Abstract

**Introduction:** The Cognitive Behavioral Analysis System of Psychotherapy (CBASP) was developed for the treatment of persistent depressive disorder (PDD), where comorbid personality disorders (PD) are common. In contrast to other PD, comorbid borderline personality disorder (BPD) is often regarded as an exclusion criterion for CBASP. In clinical settings, however, subthreshold BPD symptoms are prevalent in PDD and may not be obvious at an initial assessment prior to therapy. As data on their impact on CBASP outcome are very limited, this naturalistic study investigates BPD features in PDD and their relevance for the therapeutic outcome of a multimodal CBASP inpatient program.

**Method:** Sixty patients (37 female, mean age 38.3, SD 11.9 years) meeting DSM-5 criteria for PDD underwent a 10 weeks CBASP inpatient program. BPD features (i.e., number of fulfilled DSM-5 criteria) together with childhood maltreatment and rejection sensitivity were assessed on admission. Before and after treatment, severity of depressive symptoms was measured using the Montgomery-Asberg Depression Rating Scale (MADRS) and the Beck Depression Inventory (BDI-II). BPD symptoms were assessed using the Borderline Personality Disorder Severity Index (BPDSI-IV) and the Borderline Symptom List (BSL-23). Intercorrelations of baseline characteristics and symptom change during treatment were analyzed.

**Results:** Patients with PDD met a mean of 1.5 (SD 1.6) BPD criteria with 4 patients fulfilling ≥5 criteria. BPD symptoms and depressive symptoms showed a strong correlation, and BPD symptoms were additionally correlated with emotional abuse and rejection sensitivity. There was no association between BPD features at baseline and improvement on the MADRS, however, BPD features tended to be associated with a lower response according to the BDI-II score after 10 weeks of treatment. Furthermore, BPD symptoms (i.e., abandonment, impulsivity and affective instability) were reduced after 10 weeks of CBASP treatment.

**Discussion:** BPD symptoms are prevalent in patients with PDD and highly intertwined with the experience of depressive symptoms. In this naturalistic study in PDD, BPD features at baseline did not limit the clinical response to CBASP. Future studies may extend the spectrum of PDD to comorbid subsyndromal or even syndromal BPD in order to develop tailored psychotherapeutic treatment for these complex affective disorders.

## Introduction

Persistent depressive disorder (PDD) is a highly debilitating psychological condition characterized by interpersonal difficulties and a high rate of comorbidities (1–3). PDD is defined by a duration of depressive symptoms for a minimum of 2 years and ranging in severity from dysthymia to chronic major depression [according to DSM-5, (4)]. Findings suggest that about a third of all depressed patients develop a chronic form of depression (3, 5, 6). Compared to non-chronic forms of depression, PDD patients tend to have a significantly earlier onset and higher levels of treatment resistance (1, 2, 5).

PDD and personality disorders (PD) often co-occur and presence of both may result in higher severity and duration of depressive symptoms. Studies suggest that the prevalence of comorbid PD in PDD patients is ranging from 51.2% (7) up to 70% (8, 9). Comorbid PD has been shown to be related to higher severity of depressive symptoms (8). Vice versa, it has been indicated that an increasing duration of depressive episodes is associated with a higher frequency of PD diagnoses (10). For PDD, early onset of depression has been linked to higher rates of comorbid PD than late onset (8, 11). In addition, common factors between PDD and PD may exist and symptoms like interpersonal difficulties may overlap (12, 13).

Borderline personality disorder (BPD) is characterized by a pattern of affective instability, impulsivity and identity problems including dissociative symptoms, chronic feelings of emptiness, difficulty in controlling anger and suicidal behavior or threats (4). Evidence suggests that prevalence of BPD in depression is quite common: Comorbid BPD was found in 28% of dysthymic patients and was even more pronounced in early onset dysthymics with 42% (8). Other epidemiological studies suggest lower prevalence rates of lifetime BPD in lifetime major depressive disorder (MDD) and dysthymia, i.e., 11.5% and 16.7%, respectively (14). The other way around, mood disorders are highly prevalent in BPD patients with an 83% lifetime prevalence of comorbid MDD (15). Patients suffering from both, depression and BPD, seem to be more likely to experience a chronic course of depression (16) and BPD is a robust predictor of persistence of MDD (17). This finding persisted even after controlling for other prognostic indicators such as age at onset, treatment history or previous episodes (18).

PDD and BPD share similar risk factors and it has been suggested that high rates of comorbidity between PD and PDD might be due to shared etiological factors such as genetics, temperamental vulnerability, self-generated interpersonal stress or childhood maltreatment (CM) including invalidating educational patterns (13, 19). PDD patients experience a higher number of traumatic events during their lifetime than patients with non-chronic forms of depression (1, 3, 20). CM has been associated with severity and chronicity of depression in numerous studies (21, 22). BPD has also been associated with CM and an invalidating family environment according to Linehan's biopsychosocial model (23). Indeed, CM (including family adversity, exposure to physical and sexual abuse or neglect) has been found to be a robust BPD risk factor in a systematic review (24) and both PDD and BPD patients showed a high trauma load in the Childhood Trauma Questionnaire [CTQ, (25)]. One putative link from CM to later psychopathology may be an induced trait of rejection sensitivity, i.e., oversensitivity to and expectation of social rejection (26). Rejection sensitivity has been found to be associated with PDD (27, 28) and BPD (29). Thus, rejection sensitivity may theoretically mediate the path from CM to both PDD and BPD symptoms.

A psychotherapy approach that has been specifically developed for the treatment of PDD is the Cognitive Behavioral Analysis System of Psychotherapy [CBASP, (30)]. CBASP has been shown to be an effective treatment for PDD [e.g., (31, 32)], and the combination of CBASP with antidepressant medication is recommended by a guidance paper from the European Psychiatric Association for the treatment of PDD (33). In addition, Schramm et al. (34) found that CBASP outperformed nonspecific supportive psychotherapy in a sample of 268 PDD outpatients without antidepressant medication. CBASP may be particularly effective in patients with a history of CM (35, 36).

In terms of the effect of comorbid PD on CBASP treatment outcome, previous findings have been quite heterogeneous. Erkens et al. (12) found no significant main effect of PD (mostly cluster C PD) reducing the effectiveness of CBASP. Similarly, Maddux et al. (37) found no significant effect of comorbid PD on the outcome after receiving CBASP, pharmacotherapy or the combination. In those two and many other studies investigating the effectiveness of CBASP, the spectrum of comorbid PD has been limited by the in- and exclusion criteria applied and mainly focused on cluster C PD. That is, patients with comorbid BPD [e.g., (12, 34, 38)] or severe forms of BPD [e.g., (37, 39)] were not included. Such exclusion of BPD patients might have been due to the fact that it has been suggested that comorbid BPD symptoms could interfere with CBASP [e.g., (40, 41)]. This seems reasonable from a practitioner's point of view as there is evidence that comorbid BPD can hamper the response to treatment in episodically depressed patients [e.g., (18)]. Also, higher levels of subthreshold BPD symptoms were the only PD features that significantly affected time to remission after a 12 weeks treatment with interpersonal therapy (IPT) or pharmacotherapy in episodically depressed patients (42). Recent developments in the clinical diagnostics of PD have introduced dimensional approaches besides the categorical conceptualization (4, 43). Dimensional measures allow to assess symptoms on a subthreshold level that are apparent and may already cause impairment but do not yet justify the PD diagnosis. The introduction of dimensional models is seen as an opportunity to increase clinical utility (44) and it has been shown that subclinical BPD features in patients with mood disorder lead to higher levels of impairment (45).

The assumption of BPD diagnosis or subthreshold BPD features hampering the CBASP treatment response has not been specifically investigated in studies with PDD patients. Therefore, we formulated two research questions in our naturalistic pilot study: (1) What is the prevalence of BPD features and symptoms in a naturalistic sample of PDD inpatients seeking CBASP treatment? (2) Does the presence of BPD features in PDD patients reduce the effectiveness of a multimodal 10 weeks CBASP inpatient program in terms of less reduction of depressive symptoms? In addition, we explored the effect of a CBASP inpatient program on BPD symptoms.

## Materials and Methods

### Sample

Data for this study were collected at the Department of Psychiatry and Psychotherapy of the University Hospital, LMU Munich, Germany. Participants took part in a larger naturalistic and still on-going study assessing the effectiveness of 10 weeks disorder-specific psychotherapy (German Clinical Trial Register ID: DRKS00019821). The study was designed in accordance with the ethical standards as laid down in the 1964 Declaration of Helsinki and its subsequent amendments or comparable ethical standards and approved by the local ethics committee (Faculty of Medicine, Ludwig Maximilian University Munich, Munich, Germany, EK-No. 713-15).

**Figure 1:**
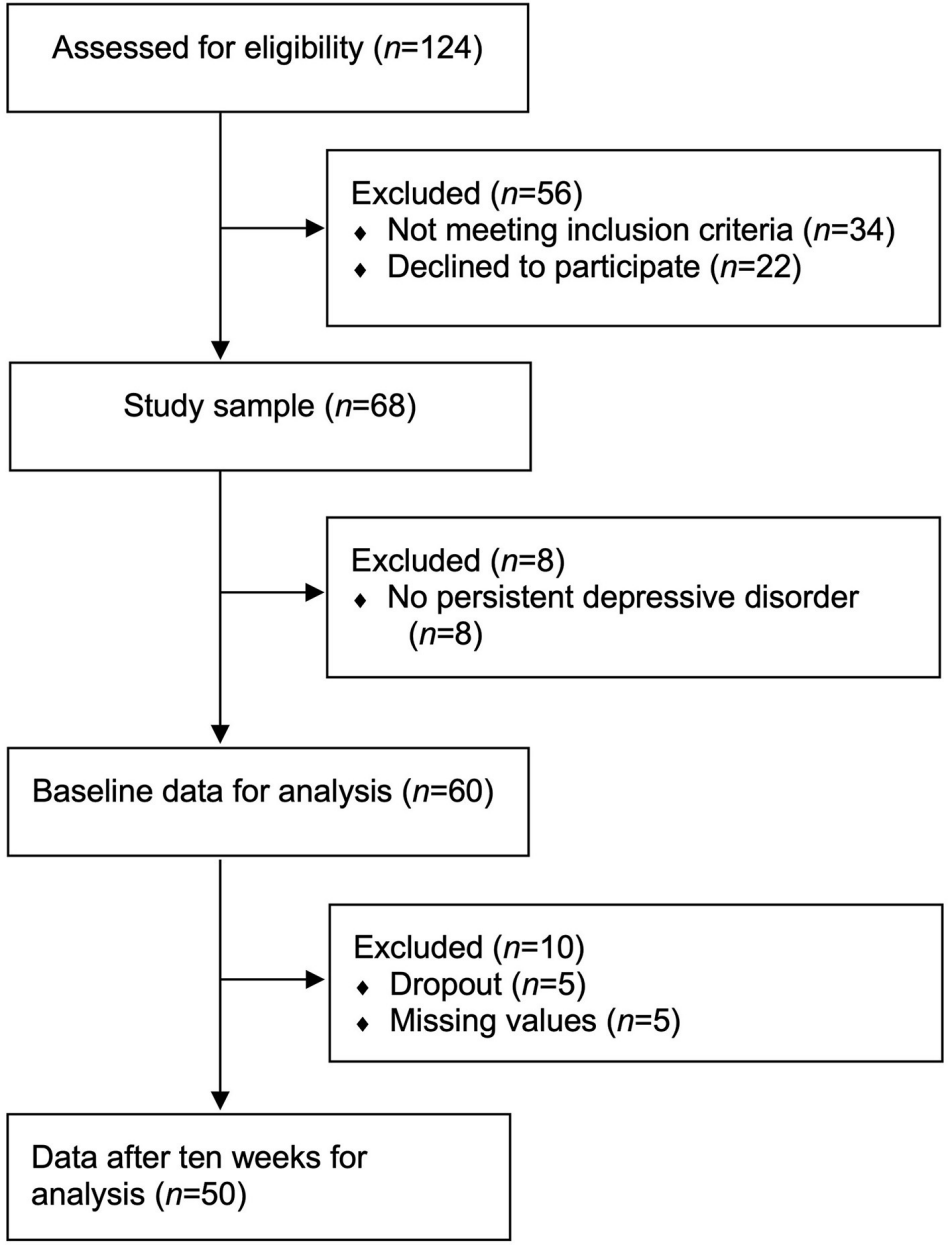
Flow chart of study participants.

After admission to the psychotherapy ward specialized in treating PDD with CBASP, 124 inpatients were screened for eligibility between June 2018 and March 2020 (see Figure 1). Patients were included if they took part in the 10 weeks CBASP program, were fluent in German and were aged between 18 and 65 years. Exclusion criteria contained acute suicidality, bipolar disorder, psychosis, a primary psychiatric diagnosis of PTSD, social phobia, panic disorder or generalized anxiety disorder, current pregnancy and/or a somatic unstable condition that needed to be treated primarily. Furthermore, patients that were admitted for a 5 weeks booster session were excluded. A diagnosis of BPD was explicitly no exclusion criterion. According to our exclusion criteria, 34 patients were not eligible after screening (5 weeks booster session: *n* = 23; bipolar disorder: *n* = 6; age >65 years: *n* = 3; non-fluent German: *n* = 2). Participants were then informed about the study and n = 22 patients decided to not take part in the study. From the remaining *n* = 68 patients that provided written informed consent prior to inclusion, *n* = 8 were excluded after the SCID-interview because they did not fulfill criteria for PDD but episodic depression. Though baseline data were collected for all 60 PDD patients, post intervention data were available only for 50 patients due to missing information (*n* = 5, 8.3%) and dropouts (*n* = 5, 8.3%).

### Treatment: CBASP Inpatient Program

All participants underwent 10 weeks of multimodal CBASP treatment following the CBASP manual (30), modified for an inpatient setting (9, 41). The CBASP program included two individual sessions per week (i.e., 20 sessions in total, 50 min each), two group sessions per week (100 + 50 min), mindfulness training (50 min), group physical therapy (50 min) and occupational therapy (100 min). In addition, patients had regular medical rounds by the attending physician as well as the senior physician and a weekly nurse-patient encounter (25 min). The whole team received regular CBASP trainings and supervision (by ELB and FP). One psychotherapist was a certified CBASP therapist (FK), the rest of the team (three medical doctors, seven psychologists) were at an advanced psychotherapy and CBASP training stage with weekly supervision.

Patients received algorithm-based psychopharmacological treatment following national guidelines for depression (46). Table 1 presents medication rates on admission and discharge.

**Table 1:** Frequency and percentage of prescribed drugs on admission and discharge (*N* = 60) and mean number of prescribed psychotropics; for CBASP dropouts medication at the date of dropout is reported.

		**Admission *n***	**Discharge *n***	***Z***	***P***
Any medication		54 (90.0%)	58 (96.7%)	2.0	0.04*
Psychotropic medication		40 (66.7%)	37 (61.7%)	1.7	0.08
Antidepressant drugs	SSRI	8 (13.3%)	6 (10.0%)	1.4	0.16
	SSNRI	12 (20.0%)	23 (38.3%)	3.3	0.001**
	Mirtazapine	6 (10.0%)	7 (11.7%)	1.0	0.32
	Bupropion	9 (15.0%)	11 (18.3%)	1.4	0.16
	Other	17 (28.3%)	10 (16.7%)	2.7	0.008**
Lithium		7 (11.7%)	6 (10.0%)	1.0	0.33
Quetiapine		12 (20.0%)	7 (11.7%)	2.2	0.03*
Aripiprazole		7 (11.7%)	11 (18.3%)	2.0	0.04*
Mean number of psychotropics		1.7 ± 1.4	1.6 ± 1.0	*t* (59) = 0.4	0.68

SSRI, selective serotonin reuptake inhibitor; SSNRI, selective serotonin noradrenaline reuptake inhibitor; Other, mainly Milnacipran, Tranylcypromine, different tricyclic antidepressants and Trazodone; p-values according to Wilcoxon rank test, (*) < 0.05, (**) < 0.01.

Furthermore, a specifically trained nurses' team offered an optional weekly group skills training (90 min) based on the Dialectical Behavioral Therapy (DBT) manual by Bohus and Wolf-Arehult (47). The DBT skills training included sessions on mindfulness, distress tolerance, emotion regulation and interpersonal effectiveness. For each patient, the number of participated group sessions was assessed. Skills group dosage ranged from 0 to 10 sessions.

### PDD and Comorbidity Assessment - BPD Features

On admission, a trained and supervised psychological research assistant assessed PDD and comorbid diagnoses with the German version of the Structured Clinical Interview for DSM-IV [SCID-I and SCID-II, (48, 49)]. In addition to SCID-I, diagnostic criteria for PDD were assessed according to DSM-5, as the German version of the DSM-5 preceded the publication of SCID-5-CV and was already available at the beginning of the study. Since its publication in 2019, the respective German interviews for DSM-5 [SCID-5-CV and SCID-5-PD, (50, 51)] were used. BPD criteria (i.e., BPD features) were rated for each participant even in the absence of BPD symptoms in the screening questionnaire.

Alongside the categorial assessment of a BPD diagnosis, SCID-II already allowed to calculate a dimensional (D-)score by summing up the scores of the nine BPD criteria (1 = absent, 2 = subthreshold, 3 = present). SCID-5-PD also offers the possibility to take subthreshold criteria into account and to characterize BPD features in more detail. However, answers are rated differently (0 = absent, 1 = subthreshold, 2 = true/threshold). As the diagnostic criteria for BPD have not changed from DSM-IV to DSM-5, we transformed SCID-II ratings to match SCID-5-PD scores. Consequently, the reported dimensional BPD score ranges between 0 and 18.

### Depressive Symptom Severity

Depressive symptoms were assessed on admission and after 10 weeks. The Montgomery–Asberg Depression Rating Scale [MADRS, (52)] was defined as primary outcome. MADRS is a clinician-based interview that assesses the severity of 10 depressive symptoms with a total score between 0 and 60. It has been found to have a high sensitivity to change (53). Interviews were conducted by the attending physicians that were trained in the Structured Interview Guide for the Montgomery-Asberg Depression Rating [SIGMA, (54)].

The Beck Depression Inventory [BDI-II, (55)] is a well-established 21-item self-report measure that assesses the severity of depressive symptoms within the last 14 days with a total score ranging from 0 to 63.

### Borderline Personality Symptom Severity

BPD symptoms were also measured at baseline and post-treatment. The Borderline Personality Disorder Severity Index – Version IV (BPDSI-IV) is a clinician-based, semi-structured interview (56, 57). It provides a quantitative index of the severity of BPD manifestation by evaluating the frequency and intensity for BPD symptoms over the course of the last 3 months. The BPDSI-IV consists of 70 items organized in nine subscales according to DSM-IV criteria. Subscales range from 0 (never) to 10 (daily), except for the subscale identity disturbance. The sum of the means of each dimension form the total score ranging from 0 to 90. The BPDSI-IV has excellent psychometric characteristics [Cronbach's alpha = 0.96; interrater reliability: *r* = 0.97, high validity and sensitivity to change, (57)].

The Borderline Symptom List [BSL-23, (58)] is a self-rating questionnaire that assesses the subjective severity of 23 BPD symptoms during the past week with a total score from 0 to 92. The BSL-23 has a high internal consistency (Cronbach's alpha = 0.93), high test-retest reliability (*r* = 0.82) and is very reliable in the diagnosis of BPD (58, 59).

### Rejection Sensitivity Questionnaire

Rejection sensitivity was assessed at baseline with the rejection sensitivity questionnaire [RSQ, (26, 60)]. Participants are asked to rate both their anxiety and their expectation to be rejected in 20 scenarios on 6-point Likert scales. Scores for each scenario are multiplied. The total score ranges from 1 to 36, with higher scores indicating higher rejection sensitivity at beginning of treatment.

### Childhood Maltreatment

The CTQ (61, 62) is a retrospective self-report measure that assesses CM on the subscales emotional abuse and neglect, physical abuse and neglect, and sexual abuse with 28 items. Participants rate whether different experiences were present during their childhood on Likert scales ranging from 1 (never true) to 5 (very often true). Subscale scores range from 5 to 25. For the German version of the CTQ a good internal consistency has been found for all subscales (alpha > 0.80) except for physical neglect (62).

### Data Analysis

We used SPSS version 25 for statistical analyzes (https://www.ibm.com/de-de/products/spss-statistics). First, baseline values were analyzed: Intercorrelations between BPD features (i.e., SCID-5-PD score for BPD), BPD symptoms and depressive symptoms were calculated with Pearson or Spearman as appropriate. Furthermore, correlations with CM and rejection sensitivity were calculated and *p*-values were false discovery rate (FDR) corrected according to Benjamini and Hochberg (63) to correct for multiple correlations. Second, dependent *t*-tests were used to compare the differences of depressive symptoms before and after therapy. Patients that did not complete 10 weeks of CBASP or had missing values after 10 weeks were excluded from these analyses. The pre-post effect sizes were calculated using Cohen's d statistic. Linear regression analyses were performed to predict the change of depressive symptoms (delta) by the BPD dimensional score. Third, change of BPD symptoms was analyzed with dependent *t*-tests. Again, *p*-values were FDR corrected. Finally, the impact of skills training participation on BPD symptoms was analyzed with Mann-Whitney-*U*-Tests (participants vs. non-participants).

## Results

### Sample

Baseline values of *n* = 60 patients were analyzed (37 females, 61.7%; mean age = 38.9, SD = 11.9). Demographic and clinical characteristics are presented in Table 2. Patients showed a variety of comorbidities including social phobia (*n* = 19, 31.7%), panic disorder/agoraphobia (*n* = 16, 26.7%), PTSD not as primary diagnosis (*n* = 6, 10.0%), pain disorder (*n* = 6, 10.0%), somatic symptom disorder (*n* = 5, 8.3%), alcohol abuse (*n* = 5, 8.3%) and binge eating disorder (*n* = 3, 5.0%). In addition, patients showed comorbidity for several PD including BPD (*n* = 4, 6.7%), avoidant (*n* = 20, 33.3%), dependent (*n* = 1, 1.7%), obsessive-compulsive (*n* = 2, 3.3%), paranoid (*n* = 2, 3.3%) and schizoid (*n* = 2, 3.3%) PD. After 10 weeks of CBASP, data from *n* = 50 patients were available (32 females, 64.0%; mean age = 39.3, SD = 12.0) because of dropouts (*n* = 5, 8.3%) and missing data (*n* = 5, 8.3%).

**Table 2:** Demographic and clinical characteristics at baseline with mean and standard deviation (SD) or number of patients (*N* = 60) and percentages.

**Demographic characteristics**	
Age at admission, years	38.9 (SD 11.9)
Female sex	37 (61.7%)
Education, years	15.5 (SD 4.2)
No degree	1 (1.7%)
In Education	3 (5.0%)
Traineeship	43 (71.7%)
College/University	13 (21.7%)
Unemployed or early retirement	25 (41.7%)
Married/with partner	18 (30.0%)
Clinical characteristics	
Persistent major depressive episode	39 (65.0%)
Intermittent major depressive episode	12 (20.0%)
with current episode	
Intermittent major depressive episode	9 (15.0%)
without current episode	
Age at onset, years	17.1 (11.2)
At least one other Axis I disorder	36 (60.0%)
At least one other Axis II disorder	24 (40.0%)
Borderline personality disorder	4 (6.7%)
SCID-5-PD BPD D-score	3.8 (SD 3.7)
Suicide attempts in the past	16 (26.7%)
Self-injury behavior in the past	25 (41.7%)
MADRS	28.0 (SD 5.4)
BDI-II	31.0 (SD 10.9)
BPDSI-IV total	16.7 (SD 8.4)
BSL-23	1.4 (SD 0.8)
CTQ Emotional abuse	14.3 (SD 6.1)
CTQ Physical abuse	7.1 (SD 3.4)
CTQ Sexual abuse	6.3 (SD 2.9)
CTQ Emotional neglect	16.0 (SD 5.1)
CTQ Physical neglect	8.8 (SD 3.5)
Rejection sensitivity questionnaire	15.0 (SD 6.0)

*SCID-5-PD BPD D-Score, SCID-5-PD dimensional score for borderline personality disorder; MADRS, Montgomery-Asberg Depression Rating Scale; BDI-II, Beck depression inventory; BPDSI-IV, borderline personality disorder severity index; BSL-23, borderline symptom list; CTQ, childhood trauma questionnaire*.

### BPD Features and Symptoms at Baseline

**Figure 2:**
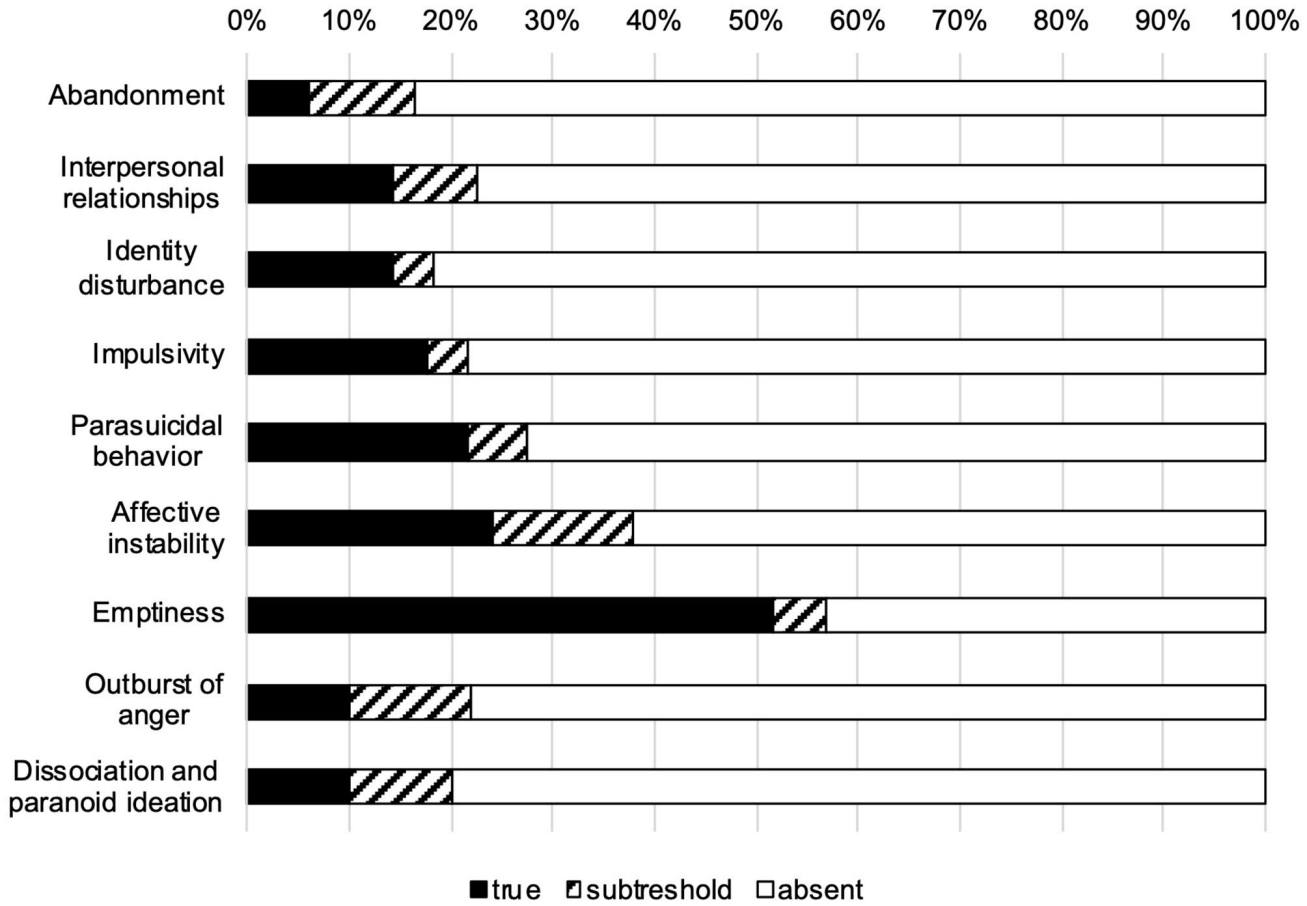
Percentage of fulfilled, subthreshold and absent borderline personality criteria (DSM-5) within patients with persistent depressive disorder (*N* = 60).

Out of *n* = 60 patients with PDD, *n* = 4 (6.7%) fulfilled the diagnosis of BPD, i.e., ≥5 criteria according to DSM-5, *n* = 3 (5.0%) fulfilled four BPD criteria. The most frequently fulfilled criteria were emptiness (criterion 7: *n* = 30), affective instability (criterion 6: *n* = 12) and parasuicidal behavior (criterion 5: *n* = 11) (see Figure 2). Patients showed a mean of 1.5 (SD = 1.6) fulfilled BPD criteria and a mean SCID-5-PD BPD dimensional score of 3.8 (SD = 3.7). BPD symptoms were present in the observer-rating BPDSI-IV (mean total score = 16.7, SD = 8.4) and self-rating measure BSL-23 [mean = 1.4, SD = 0.8; moderate severity according to (59)]. Patients reported depressive symptoms at baseline (MADRS: mean = 28.0, SD = 5.4; BDI: mean = 31.0, SD = 10.9). Measurements for BPD features and symptoms showed a high intercorrelation of self- and observer-rating (see Table 3). Furthermore, total BPDSI-IV and BSL-23 correlated significantly with depressive symptoms.

**Table 3:** Intercorrelation (Pearson or Spearman as appropriate) of borderline personality disorder (BPD) features, BPD symptoms and depressive symptoms at baseline, *p*-values are false discovery rate corrected (FDR).

	**SCID-5-PD**	**BPDSI-IV total**		**BSL-23**		**MADRS**		**BDI-II**	
	**BPD D-score**	***r***	***p_***FDR***_***	***r***	***p_***FDR***_***	***r***	***p_***FDR***_***	***r***	***p_***FDR***_***
SCID-5-PD BPD D-Score	**–**	0.53	0.001**	0.52	<0.001***	0.16	0.23	0.06	0.67
BPDSI-IV total	**–**	**–**	**–**	0.66	<0.001***	0.23	0.09	0.41	0.001**
BSL-23	**–**	**–**	**–**	**–**	**–**	0.42	0.04**	0.67	<0.001***
MADRS	**–**	**–**	**–**	**–**	**–**	**–**	**–**	0.48	<0.001***
BDI-II	**–**	**–**	**–**	**–**	**–**	**–**	**–**	**–**	**–**

SCID-5-PD BPD D-Score, SCID-5-PD dimensional score for borderline personality disorder; BPDSI-IV, borderline personality disorder severity index; BSL-23, borderline symptom list; MADRS, Montgomery-Asberg Depression Rating Scale; BDI-II, Beck depression inventory; (**) < 0.01, (***) < 0.001.

Patients reported a history of CM including emotional abuse [mean = 14.3, SD = 6.1, 55.0% at least moderate to severe as defined by (61)], physical abuse (mean = 7.1, SD = 3.5, 20.0% at least moderate to severe), sexual abuse (mean = 6.2, SD = 2.9, 18.3% at least moderate to severe), emotional neglect (mean = 16.0, SD = 5.1, 66.7% at least moderate to severe) and physical neglect (mean = 8.8, SD = 3.5, 30.0% at least moderate to severe). Emotional abuse and physical neglect showed a significant correlation with BPD symptoms and subjective depressive symptoms at baseline (see Table 4). In addition, rejection sensitivity (mean = 15.0, SD = 6.0) was significantly correlated with self-reported BPD and depressive symptoms at baseline.

**Table 4:** Correlation (Pearson or Spearman as appropriate) of borderline personality disorder (BPD) features, BPD symptoms and depressive symptoms with trauma history and rejection sensitivity, *p*-values are false discovery rate corrected (FDR) for the number of subscales.

	**SCID-5-PD** **BPD D-score**		**BPDSI-IV total**		**BSL-23**		**MADRS**		**BDI-II**	
	***r***	***p_***FDR***_***	***r***	***p_***FDR***_***	***r***	***p_***FDR***_***	***r***	***p_***FDR***_***	***r***	***p_***FDR***_***
**CTQ**										
Emotional abuse	0.29	0.31	0.35	0.009**	0.52	0.003**	0.05	0.69	0.49	0.003**
Physical abuse	0.14	0.35	0.19	0.23	0.35	0.02*	0.06	0.67	0.34	0.002**
Sexual abuse	0.06	0.81	0.04	0.78	0.22	0.40	0.13	0.40	0.14	0.40
Emotional neglect	0.12	0.37	0.11	0.40	0.21	0.27	0.18	0.27	0.20	0.27
Physical neglect	0.23	0.13	0.33	0.02*	0.37	0.02*	0.03	0.80	0.22	0.15
**RSQ**	0.23	0.14	0.22	0.09	0.51	0.003**	0.28	0.04*	0.53	0.003**

SCID-5-PD BPD D-Score, SCID-5-PD dimensional score for borderline personality disorder; BPDSI-IV, borderline personality disorder severity index; BSL-23, borderline symptom list; MADRS, Montgomery-Asberg Depression Rating Scale; BDI-II, Beck depression inventory; CTQ, childhood trauma questionnaire; RSQ, rejection sensitivity questionnaire; (*) < 0.05, (**) < 0.01.

### Effects of 10 Weeks CBASP and Impact of BPD Features

Depressive symptoms were reduced after 10 weeks of CBASP both on observer-rated (MADRS: *t*(49) = 9.12, p_FDR_ = 0.007, *d* = −1.41) and self-reported level (BDI-II: *t*(49) = 5.59, *p*_FDR_ =.007, *d* = −0.90; see Table 5). *N* = 7 patients (14.0%) showed a full MADRS response (Delta MADRS ≥ 50%) and *n* = 22 patients (44.0%) a partial response (Delta MADRS 25–50%). *N* = 3 patients (6.0%) reached remission on the MADRS whereas *n* = 3 (6.0%) showed a deterioration of symptoms. Regarding BDI-II, *n* = 11 patients (22.0%) showed a full response (Delta BDI-II ≥ 50%) and *n* = 14 (28.0%) patients showed a partial response (Delta BDI-II 25–50%). *N* = 5 patients (10.0%) reached remission (BDI-II ≤ 10) whereas *n* = 11 patients (22.0%) reported deterioration of symptoms (BDI-II increase). Baseline symptom severity of patients that dropped out or had missing data did not significantly differ from CBASP completers [MADRS: *t*(58) = 0.27, *p* = 0.79, *d* = 0.10; BDI-II: *t*(58) = 0.76, *p* = 0.45, *d* = 0.26; BPDSI-IV total: *t*(58) = 1.47, *p* = 0.15, *d* = 0.51; BSL-23: (58) = 0.91, *p* = 0.37, *d* = −0.32].

**Table 5:** Mean and standard deviation of depressive symptoms and borderline personality symptoms before and after 10 weeks of CBASP therapy for *n* = 50 patients (results of *t*-test or Wilcoxon as appropriate), *p*-values are false discovery rate (FDR) corrected.

	**Before**	**After**	***t(49), Z***	***p***	***p_***FDR***_***	***d (CI95%)***
MADRS	27.9 (5.4)	19.6 (6.3)	9.12	<0.001***	0.007**	−1.41 (−1.84 to −0.97)
BDI-II	30.5 (11.0)	23.6 (13.3)	5.59	<0.001***	0.007**	−0.90 (−1.31 to −0.49)
BPDSI-IV total	15.3 (7.4)	13.0 (7.2)	2.89	0.006**	0.02*	−0.51 (−0.91 to −0.11)
1. Abandonment	1.4 (1.3)	0.9 (1.0)	−2.61	0.009**	0.02*	−0.34 (−0.74 to 0.05)
2. Interpersonal relationships	0.9 (0.8)	0.8 (0.9)	−0.077	0.94	0.94	−0.02 (−0.41 to 0.37)
3. Identity disturbance	1.1 (1.3)	0.9 (1.0)	−1.27	0.21	0.27	−0.19 (−0.58 to 0.20)
4. Impulsivity	0.5 (0.5)	0.2 (0.4)	−3.10	0.002**	0.009**	−0.43 (−0.83 to −0.04)
5. Parasuicidal behavior	0.7 (0.7)	0.6 (0.6)	−0.70	0.48	0.52	−0.08 (−0.47 to 0.32)
6. Affective instability	5.0 (2.4)	4.0 (2.2)	2.89	0.006**	0.02*	−0.40 (−0.79 to 0.00)
7. Emptiness	4.0 (2.4)	3.3 (2.5)	2.10	0.04*	0.07	−0.30 (−0.70 to 0.09)
8. Outburst of anger	1.1 (1.2)	1.0 (1.1)	−0.72	0.47	0.52	−0.12 (−0.51 to 0.27)
9. Dissociation and paranoid ideation	1.4 (1.0)	1.2 (1.4)	−1.52	0.13	0.19	−0.23 (−0.63 to 0.16)
BSL-23	1.3 (0.7)	1.2 (0.9)	1.96	0.06	0.10	−0.31 (−0.70 to 0.09)

MADRS, Montgomery-Asberg Depression Rating Scale; BDI-II, Beck depression inventory; BPDSI-IV, borderline personality disorder severity index; BSL-23, borderline symptom list; (*) < 0.05, (**) < 0.01, (***) < 0.001.

**Figure 3:**
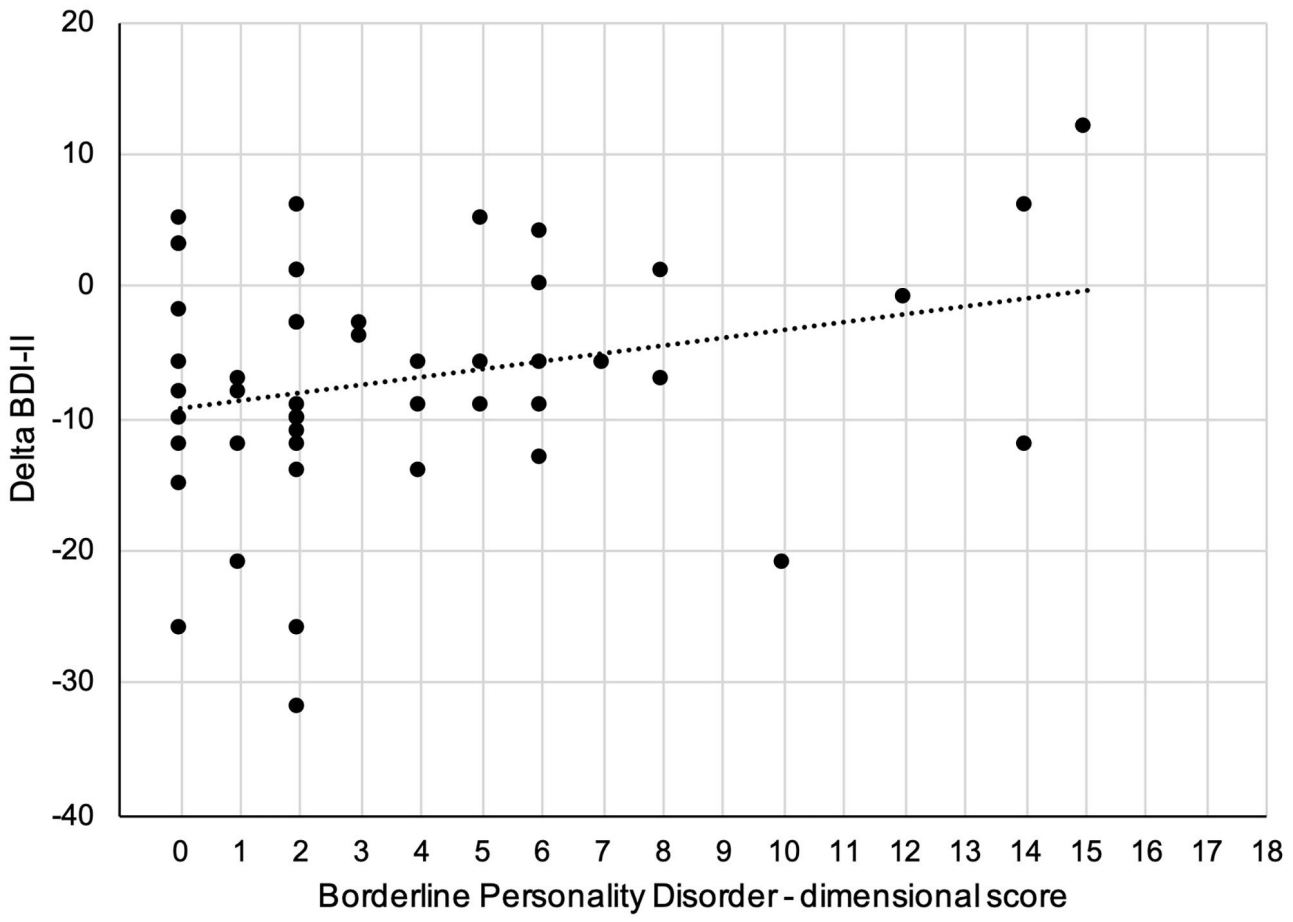
Scatter plot of the relationship between dimensional assessed borderline personality features and change of self-rated depression scores (BDI-II: Beck depression inventory) after 10 weeks.

Linear regression analysis with the SCID-5-PD BPD dimensional score as independent variable and change of MADRS as dependent variable found that BPD features did not predict change of MADRS [Beta = −0.04, *t*(47) = 0.24, *p* = 0.81]. However, when using BDI-II as dependent variable, a trend was found for an association between BPD features at baseline and a smaller reduction of BDI-II scores after 10 weeks of CBASP treatment [Beta = 0.26, *t*(47) = 1.85, *p* = 0.07; see Figure 3].

### Explorative Analysis of Change of BPD Symptoms

The total score of observer-rated BPD symptoms significantly decreased after 10 weeks [BPDSI-IV: *t*(49) = 2.89, p_FDR_ = 0.02, *d* = −0.51] with significant reductions in the subscales abandonment, impulsivity, affective instability, and a trend for emptiness (see Table 5). There was a statistical trend for a reduction of self-reported BPD symptoms after 10 weeks of CBASP [BSL-23: *t*(49) = 1.96, p_FDR_ = 0.10, *d* = −0.31]. When controlling for change of MADRS, the reduction of impulsivity remained significant (p_FDR_ = 0.047).

Thirty-nine out of 50 patients participated in at least one session of DBT skills training (mean session number = 5.2, SD = 3.4) that may have contributed to a reduction of BPD symptoms. Patients that attended DBT skills training did not differ from patients without DBT skills training regarding MADRS, BDI-II, or BSL-23 at baseline (*p* > 0.10). However, there was a trend that patients that participated in DBT skills training reported more BPD symptoms at baseline in the clinician-based interview (BPDSI-IV total: *Z* = 1.91, *p* = 0.06, *d* = 0.64). Furthermore, there was a trend that patients with DBT skills training showed a stronger BPDSI-IV reduction than patients without skills training (*Z* = 1.72, *p* = 0.09, *d* = −0.58).

## Discussion

The aim of this naturalistic study was the assessment of BPD features and symptoms in a sample of PDD inpatients undergoing a 10 weeks multimodal CBASP program and their impact on the therapeutic outcome. We found that BPD symptoms were prevalent in PDD patients and highly intertwined with self-reported depressive symptoms and a history of emotional abuse. There was a trend that BPD features were associated with a smaller reduction of self-reported but not observer-rated depression scores after receiving CBASP. However, BPD features or symptoms did not evidently limit the effectiveness of the inpatient program, and BPD symptoms partially improved after 10 weeks. To our knowledge, this is the first study investigating the effect of BPD features on treatment outcome in patients with PDD.

At baseline, we found a particular high prevalence of emptiness and affective instability in PDD. Other typical BPD features like impulsive behavior, parasuicidal behavior and outbursts of anger were less prevalent, yet present. Naturally, prevalence of BPD features and symptoms was higher in our sample than the prevalence of fully diagnosed BPD due to the dimensional approach used here. Additionally, PDD patients showed a high rate of comorbidity with social phobia and avoidant PD. This finding corresponds with other results of PDD inpatients (9, 64) and outpatients (12) that showed a high comorbidity with anxiety disorders. However, these studies did not specifically assess BPD features and symptoms. BPD symptoms seem to be highly intertwined with depressive symptoms as the marked correlations between self-reported depression and self-reported BPD symptoms suggest. This result confirms previous findings that patients with BPD and depression tend to report higher depression scores than depressed patients without comorbid BPD (65). It has been suggested that the subjective experience of depression in BPD may be more severe and intense (65). However, BPD features (as measured by SCID-5-PD BPD dimensional score) did not correlate significantly with depressive symptoms in our sample whereas self-reported BPD symptoms did (as measured by BSL-23). This may be due to our small sample size and reduced variance of the dimensional score. Interestingly, previous research found that patients with current depression showed more BPD features than patients with remitted depression (66) and BPD seemed to be associated with a longer persistence of depressive symptoms (16, 17). Taken together, BPD features and symptoms may augment the experience of depressive symptoms and contribute to a chronic course of depression.

In addition, BPD symptoms were associated with a history of emotional abuse in our sample. Indeed, CM has been found to be a risk factor for both PDD and BPD [e.g., (1, 24)]. Foxhall et al. (29) have proposed rejection sensitivity to be linked to both CM (i.e., particularly emotional abuse and neglect) and to BPD. Similarly, rejection sensitivity appears to be elevated in PDD compared to healthy controls (27, 28) and was correlated not only with BPD symptoms but also with self-reported depression. Rejection sensitivity may in fact be a mediating factor between childhood adversity and later psychopathology though this hypothesis still needs to be tested in larger cross-diagnostic studies (29).

Depressive symptoms were reduced after 10 weeks of CBASP in our sample. The general effectiveness of CBASP for PDD has been shown in numerous studies (31, 32, 34). However, our response and remission rates were lower than in comparable CBASP inpatient studies (9, 64, 67) with a longer treatment duration of 12 weeks compared to our program. As CBASP has been specifically developed for the treatment of PDD, there is basically no data on the effectiveness of CBASP in other psychiatric disorders including BPD. Patients with comorbid BPD have even been excluded in the majority of randomized controlled CBASP trials. From a practitioner's point of view, BPD features, like impulsivity and self-harm, could potentially interfere with CBASP as they shift the therapeutic focus away from CBASP toward emotion and impulse regulation. Indeed, our results suggest that the presence of BPD features may reduce the effectiveness of our inpatient program regarding self-reported depressive symptoms but not observer-rated depressive symptoms. The discrepancy of interview-based and self-report instruments for depression is well-known and clinician-rated instruments result in higher effect sizes for treatment outcome (68). It has been suggested that self-report instruments are less sensitive to change than observer-ratings (68) and that patients with BPD subjectively experience depression more intensely (65). In general, this discrepancy of self and observer perception may lead to the problem that patients might perceive CBASP less effective than therapists do, which could result in misunderstandings and invalidating experiences for the patient. Yet, if therapists are aware of this issue, they could address and clarify this discrepancy.

Interestingly, we found a reduction of BPD symptoms in the subscales abandonment, impulsivity and affective instability. In addition, feelings of emptiness seemed to be reduced after 10 weeks. A reduction of depressive symptoms may lead to an alleviation of BPD symptoms due to the observed intercorrelation. However, the reduction of impulsivity remained significant when controlling for the change of MADRS. Another possible explanation for the reduction of BPD symptoms is the opportunity to attend DBT skills training in addition to the CBASP program. DBT skills training particularly addresses difficulties in emotion and stress regulation by teaching skills in stress tolerance, interpersonal behavior and mindfulness (47, 69). DBT has been shown to be effective in the treatment of patients with BPD (70). However, there was only a limited dosage of group sessions that patients could attend during their stay (with a maximum of 10 sessions) and it was not possible to undergo all the modules included in the DBT skills training (47). Also, most inpatients in our sample attended a limited number of group sessions. Another explanation for the reduction of some of the self-reported BPD symptoms could be that CBASP elements contributed to the reduction in a similar way as interventions from other evidence-based therapies for BPD do. Storebø et al. (70) state that the focus on the therapeutic relationship is a common element in all disorder-specific therapies addressing BPD. The therapeutic alliance is also one of the core elements of CBASP, as disciplined personal involvement (DPI) of the therapist through contingent personal responsivity (CPR) and the interpersonal discrimination exercise (IDE) gives the patient the opportunity to experience and perceive a new interpersonal reality within the session (30, 71).

Further studies are needed in order to disentangle specific actions and identify effective components across therapies. In fact, prospective trials investigating a specific psychotherapy in a cross-diagnostic spectrum are generally lacking. Thus, studies as ours investigating the efficacy of a distinct psychotherapeutic approach in a spectrum of the primary disorder and its comorbidity (e.g., PD features) may be an approximation toward this issue. Recent developments in psychotherapy research focus more and more on individually tailored treatments that address the individual patient's needs (72). Adjusting treatment via a modular approach bears the opportunity to combine evidence-based therapeutic strategies for PDD patients that show a great variety of comorbidities (1, 41). Future research is needed to investigate possible advantages of modular treatments.

This is, to our knowledge, the first study assessing the effect of BPD features on CBASP outcome in a naturalistic sample of PDD inpatient. Therefore, the lack of a control group and randomization are clear limitations of the study. Besides CBASP, patients may have had a benefit from a variety of unspecific factors (e.g., inpatient setting with daily routines, high amount of interpersonal support, medication, voluntary participation in DBT skills training). Furthermore, we assessed BPD features (i.e., DSM criteria) only at admission and we did not assess long-term outcome after discharge of a rather short treatment program of 10 weeks. BPD and PDD may show a conceptual overlap [e.g., (19, 73)] represented by a poor divergent validity and high intercorrelation of self-report instruments for depression and BPD [e.g., (58)]. Additionally, the sample of patients, which met the criteria for BPD, was small, i.e., on a case series level. Also, a (self-)selection bias may have occurred as patients with predominant BPD features and symptoms may either seek BPD directed therapy or have BPD directed treatment recommended by clinicians. Therefore, a randomized trial that compares CBASP with a BPD directed treatment in a larger sample and with patients suffering from higher severity of BPD features would be essential to support treatment decisions.

In sum, our clinical experience and the results of this study suggest a general feasibility of CBASP in patients with BPD features in an inpatient setting. Nevertheless, several requirements need to be met like a sufficient ability to regulate high-risk suicidal and self-injuring behavior that may disrupt the regular CBASP therapy process. Similarly, other therapy-hampering patterns as fluctuating motivation, difficulties in recall and sudden dissociative states may need to be addressed specifically. The use of a therapy contract (analogous to DBT) together with regular inter- and supervision in an interdisciplinary team with broad psychotherapeutic expertise has proven to be extremely valuable. Therefore, successively combined approaches, i.e., evidence-based treatment for BPD (such as DBT Stage 1) followed by CBASP, has been promising in our experience. A thorough investigation whether attending DBT or other evidence-based BPD therapies beforehand could increase the effectiveness of CBASP for those patients would be interesting.

## Conclusion

Prevalent BPD features and BPD symptoms contribute to the symptom burden of patients with PDD and may affect the subjective CBASP outcome. Therefore, therapists should pay attention to the presence of subsyndromal BPD in PDD patients participating in a CBASP program. Nevertheless, CBASP has been found to be a feasible treatment option for PDD with BPD features. Our findings suggest that it might not be necessary to exclude these patients from receiving CBASP *per se* as there is a benefit in terms of reduction of depressive symptoms and even BPD symptoms. Strategies to regulate emotions and impulsivity may be necessary to enhance its therapeutic effectiveness. In order to even better tailor treatment to the individual PDD patient, future CBASP studies may include patients with comorbid subsyndromal and syndromal BPD.

## Data Availability Statement

The raw data supporting the conclusions of this article will be made available by the authors, without undue reservation.

## Ethics Statement

The studies involving human participants were reviewed and approved by Ethics committee Faculty of Medicine Ludwig Maximilian University Munich Munich, Germany EK-No. 713-15. The patients/participants provided their written informed consent to participate in this study.

## Author Contributions

MR, FK, BB, RM, AJ, and FP designed research. MR, FK, FG-W, TN-M, KF, BB, SG, and E-LB analyzed and interpreted data. MR, FK, and FP wrote first draft of manuscript. All authors revised the work critically and approved the final manuscript and agree to be accountable for the content of the work.

## Conflict of Interest

FP is a member of the European Scientific Advisory Board of Brainsway Inc., Jerusalem, Israel, and has received speaker's honoraria from Mag&More GmbH and the neuroCare Group. His lab has received support with equipment from neuroConn GmbH, Ilmenau, Germany, and Mag&More GmbH and Brainsway Inc., Jerusalem, Israel. The remaining authors declare that the research was conducted in the absence of any commercial or financial relationships that could be construed as a potential conflict of interest.
